# Design and content validation of AUTIVA: an ecobiopsychosocial instrument for childhood autism

**DOI:** 10.3389/fpubh.2026.1768438

**Published:** 2026-07-22

**Authors:** Ruben Castillo-Ortega, Carol Hewstone-García, Gladys Belmar-Riquelme, Vannia Jara-Mella

**Affiliations:** 1 Facultad de Cs. Para la Rehabilitación y Calidad de Vida, Universidad San Sebastián, Sede Valdivia, Chile; 2 Vicerrectoría de Vinculación con el Medio, Universidad San Sebastián, Sede Valdivia, Chile; 3 Facultad de Educación, Universidad San Sebastián, Sede Valdivia, Chile

**Keywords:** autism spectrum disorder, autistic disorder, biopsychosocial models, content validity, health equity, health planning, Latin America, public health surveillance

## Abstract

There are no community-based surveys for the development or assessment of public policies with an ecobiopsychosocial approach for autistic children. This article reports the development of an instrument assessing characteristics of Chilean autistic children, in order to understand how autism manifests in different environments. This study consisted of two stages: Firstly, after a comprehensive literature review, an instrument was designed by health and education professionals alongside autistic individuals and primary caregivers. Secondly, content validation was performed using the Content Validity Index, through the calculation of the Lawshe Content Validity Ratio. Sixty questions were validated, distributed across six subscales: “General Health Profile” obtained a Content Validity of 0.94; The subscales “Sex and Gender,” “Autism Diagnosis and Concomitant Pathologies,” “Family Income and the Impact of Autism on Socioeconomic Status,” “Diet of the Autistic Person,” and “Therapies and Schooling” obtained Content Validity Indexes of 0.89; 0.98; 1.0; 0.96; and 1.0, respectively. The Content Validity Index for the full instrument was 0.96. The survey presented demonstrated initial evidence of content validity and may serve as a basis for future psychometric validation. It allows for the assessment of this population in various life aspects, and offers great potential for creating public care policies regarding this community. As this study represents an initial content validation phase, future research should assess construct validity, criterion-related validity, reliability, and cross-cultural applicability.

## 1 Introduction

Autism is defined as a neurodevelopmental condition that manifests in diverse and multidimensional ways (1). Evidence shows that prevalence worldwide has increased in the past decades, according to data provided by the World Health Organization (WHO) (2). In Latin America, childhood autism should be understood within an ecological framework in which individual characteristics interact with family, school, health-service, socioeconomic, cultural, and policy environments. At the microsystem level, the child’s experience is shaped by family caregiving, daily routines, nutrition, and immediate support (3). At the mesosystem level, it depends on the interaction between families, schools, health professionals, and therapeutic services. At the exosystem level, access is influenced by caregiver employment, service availability, waiting lists, transportation, and out-of-pocket costs (4). At the macrosystem level, disability legislation, inclusive education policies, stigma, cultural beliefs, and socioeconomic inequality shape recognition and support (5). The chronosystem adds a temporal dimension, highlighting how diagnostic delays, school transitions, therapy continuity, and policy changes affect autistic children and their families over time. This perspective is particularly relevant in Latin America, where evidence points to limited prevalence data, uneven service access, and persistent barriers to comprehensive care.

In Chile, information regarding autistic children remains limited, and both prevalence and disability burden are not yet fully understood (6, 7), directly affecting the development of public policies. Given the complexity and heterogeneity of autistic children’s needs, a comprehensive and interdisciplinary approach is required to understand the multiple dimensions involved in their development and contexts. This perspective goes beyond the biopsychosocial approach, which considers the anatomical and physiological conditions of the individual together with the social environments that influence them (8, 9), and supports the transition toward an ecobiopsychosocial model.

Although diagnostic instruments such as the Modified Checklist for Autism in Toddlers-Revised (M-CHAT-R/F), the Autism Diagnostic Observation Schedule, Second Edition (ADOS-2) and the Autism Mental Status Exam (AMSE) (10 – 12) are internationally recognized and highly valuable for screening, clinical observation and diagnostic support, their purpose differs from that of AUTIVA. These tools are primarily designed to identify autistic traits, support diagnostic classification, or guide clinical decision-making according to established diagnostic criteria. AUTIVA, by contrast, is not intended to diagnose autism or to replace standardized diagnostic procedures. Its purpose is to characterize the broader living conditions, support needs, barriers, strengths and contextual determinants that shape the everyday experiences of autistic children and their families. In this sense, AUTIVA should be understood as a complementary public health and research instrument, designed to generate multidimensional information that may inform local surveillance, service planning and policy development.

The ecobiopsychosocial approach adopted in AUTIVA builds on, but extends beyond, the traditional biopsychosocial model. While the biopsychosocial model represented a major shift from reductionist biomedical explanations by recognizing that health and development are shaped by the interaction of biological, psychological, and social dimensions, its application to autism may still remain centered on the individual and their immediate characteristics. The transition toward an ecobiopsychosocial model responds to the need to expand health assessment beyond these individual dimensions by incorporating ecological and environmental determinants as integral components of health, wellbeing, and behavior change (13 – 15).

Drawing on Bronfenbrenner’s ecological systems theory, AUTIVA situates the autistic child within nested and interacting systems, assuming that the experience of autism is shaped not only by clinical or developmental characteristics, but also by family organization, school inclusion, access to therapies, socioeconomic resources, caregiver burden, institutional responses, and broader cultural and policy frameworks. Therefore, the “eco” dimension does not simply add more variables to the biopsychosocial model; rather, it provides an organizing structure for understanding how autism-related needs emerge, are supported, or are intensified within specific environments. From this perspective, therapy access is not only a clinical issue, but also a family, economic, and territorial issue; school participation is not only an educational variable, but also reflects institutional inclusion, peer relationships, and support systems; and nutritional patterns may be shaped by sensory characteristics, caregiver routines, health guidance, and household resources. In the Chilean context, where information on autistic children remains limited, this perspective is particularly relevant for developing instruments capable of capturing contextual dimensions that are necessary to inform research, strengthen public policies, and complement clinical and psychological assessments with information about the individual’s structural, personal, and social reality (10–13).

To our knowledge, no instruments are currently available in Latin America to comprehensively characterize autistic children from an ecobiopsychosocial perspective for public health and policy-planning purposes. This article describes the development of the AUTIVA instrument, its expert-informed content validation, and its potential applications in research and public health as a tool to assess and characterize the ecobiopsychosocial profile of autistic children and adolescents.

## 2 Materials and methods

### 2.1 Study design

**Figure 1:**
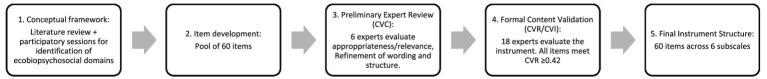
Development and validation process of the AUTIVA instrument. The figure summarizes the five methodological stages: (1) construction of the conceptual framework, (2) item development, (3) preliminary expert assessment using CVC, (4) formal content validation using CVR and CVI, and (5) final structuring of the ecobiopsychosocial domains.

As shown on Figure 1, this study followed a two-stage instrument development design aimed at constructing and validating the content of AUTIVA, an ecobiopsychosocial assessment tool for autistic children. Stage 1 consisted of the conceptualization and generation of items through literature review and participatory processes with caregivers and professionals. For Stage 2, and in order to strengthen methodological rigor, content validation followed a dual approach. The preliminary evaluation relied on Hernández-Nieto’s Coefficient of Content Validity (CVC) to identify issues related to clarity, semantic precision and item relevance. After iterative refinement, the instrument underwent formal validation using Lawshe’s CVR and the CVI, which quantify consensus on item essentiality and conceptual adequacy. The joint application of CVC and CVR/CVI ensured both linguistic quality and theoretical robustness of the final instrument.

Accordingly, the scope of this manuscript is limited to instrument development and expert-based content validity. Evidence of construct validity, criterion-related validity, and reliability will require subsequent empirical testing in larger and more diverse samples.

### 2.2 Conceptual framework and item development (stage 1)

**Figure 2:**
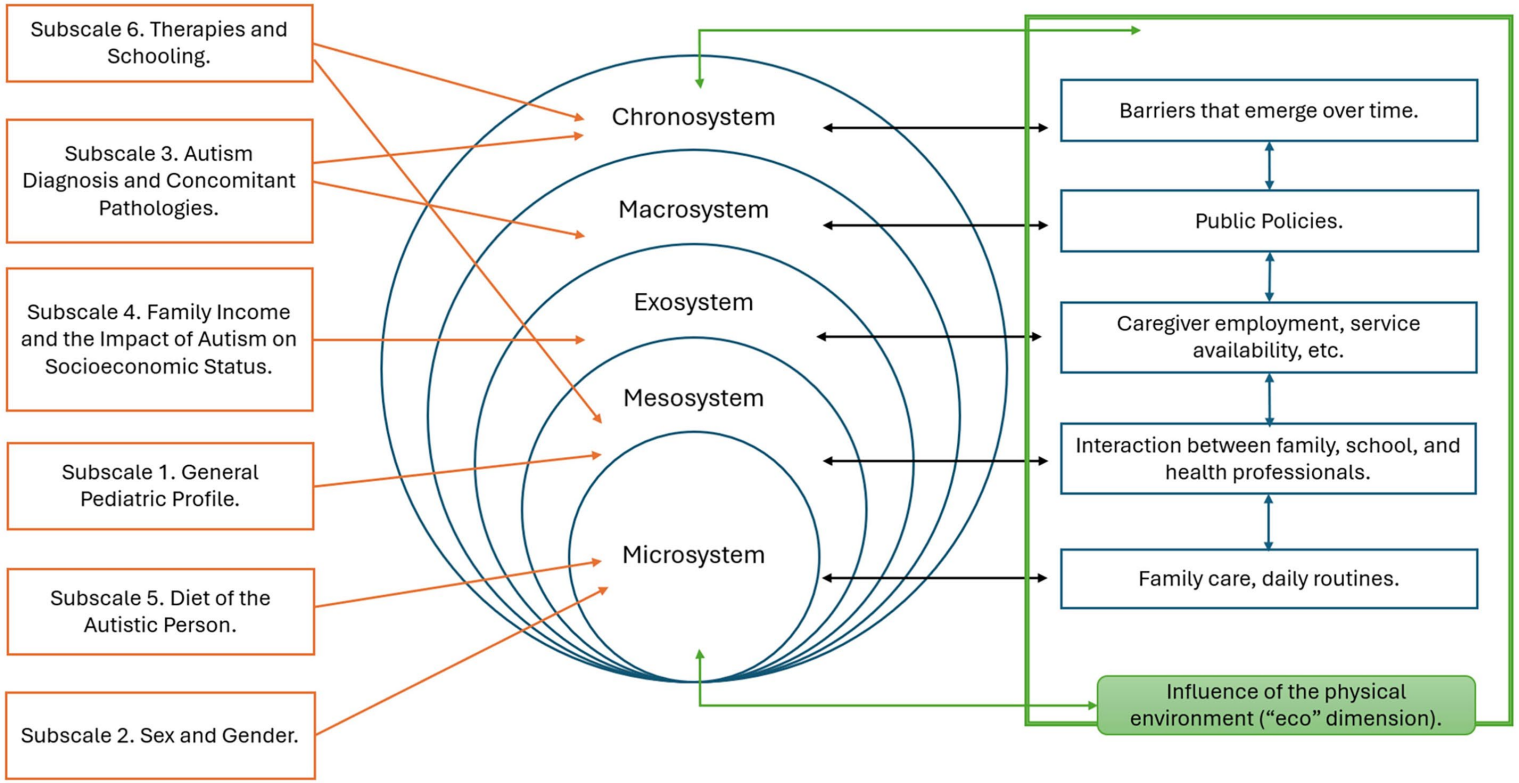
Ecological operationalization of AUTIVA subscales within the ecobiopsychosocial framework for childhood autism.

The initial version of AUTIVA was built upon an ecobiopsychosocial framework that integrates clinical, developmental, nutritional, educational, therapeutic, and socioeconomic determinants relevant to autistic children (See Figure 2). Thus, a narrative literature review was conducted to identify constructs commonly used in autism assessment tools, with emphasis on multidimensional and context-sensitive approaches. To complement the literature review, five participatory sessions were conducted, each lasting 3 h. These sessions included eight participants: four researchers with expertise in autism —one PhD in Public Health, one PhD in Special Education, one nutritionist specialized in food selectivity, and one occupational therapist with several years of experience in autism intervention—, three primary caregivers of autistic children and adolescents, and one autistic adult requiring level 1 support. These sessions helped identify context-specific variables—such as financial barriers, therapy discontinuities, nutritional challenges, and school inclusion experiences—that are underrepresented in existing instruments but are highly relevant in Chile. The decision-making process for constructing the instrument followed a qualitative-to-quantitative logic. First, the experiences, perceptions, and priorities expressed during the participatory sessions were organized according to the ecological framework guiding AUTIVA. Then, these qualitative inputs were translated into measurable items, ensuring that each question corresponded to one or more relevant ecological levels and to the broader ecobiopsychosocial domains of the instrument. Particular attention was given to the perspectives of primary caregiver mothers and the autistic adult participant, whose contributions informed the wording, syntax, order, and applicability of the questions. This process was especially important because AUTIVA was designed to be administered mainly to primary caregivers of autistic children and adolescents; therefore, the instrument required clear, accessible, and contextually meaningful language.

The original questionnaire was developed and administered in Spanish; item labels and response formats are presented here in their English-translated form for clarity.

For replication purposes, Supplementary material S1 includes a detailed breakdown of each AUTIVA subscale and the rationale behind their guiding questions.

### 2.3 Content evaluation (stage 2)

The content evaluation process consisted of two sequential procedures that served different but complementary purposes. Hernández-Nieto’s Content Validity Coefficient (CVC) was used as a preliminary refinement strategy focused on clarity, comprehensibility, appropriateness, relevance, logical organization, and sufficiency of the initial item pool. This stage helped identify potential wording problems, semantic ambiguities, and organizational issues before ethics approval and field administration. Lawshe’s Content Validity Ratio (CVR) and the Content Validity Index (CVI) were subsequently used as the formal expert-based content validation procedure. Unlike the preliminary CVC stage, which focused mainly on clarity and relevance, the CVR assessed whether each item was considered essential for evaluating the ecobiopsychosocial construct and domains represented by AUTIVA., while the CVI provided an overall estimate of expert agreement regarding the content validity of the instrument. Thus, CVC was used for refinement, whereas CVR/CVI was used for formal content validation.

#### 2.3.1 Preliminary content evaluation (CVC)

Following the creation of the initial version of AUTIVA and prior to submission to the Ethics Committee, an initial content evaluation was conducted by a panel of six experts. These professionals were selected based on their specialty, academic degree, and at least 5 years of field experience with autism. The panel included professionals from psychology, nutrition, special education, and social work.

The experts individually completed a structured evaluation form with closed-ended questions designed to assess the instrument’s appropriateness and relevance. Items were rated using a 5-point Likert scale, where 1 = strongly disagree, 2 = disagree, 3 = not sure, 4 = agree, and 5 = strongly agree. The experts also evaluated the logical sequence and distribution of the questions, as well as the sufficiency of the items to collect the intended information. In addition, open-ended questions allowed them to provide qualitative observations and suggestions regarding wording, clarity, structure, and content coverage.

Subsequently, Hernández-Nieto’s CVC (14) was calculated for each evaluated item. The coefficient was estimated using the formula: Eq. 1: CVC = (Mx/Vmax) - Pei, where Mx represents the item’s mean score given by the experts; Vmax, the maximum score the item could achieve; and Pei, the error assigned to each item (See Table 1). Based on the experts’ quantitative ratings and qualitative comments, the research team revised the wording and ordering of selected items while preserving the core domains identified during the conceptual development phase.

**Table 1:** Instrument content validity coefficient.

Variable	Item	Average	SD	CVC
Appropriateness	1	4.5	0.37	0.9
	2	4.7	0.43	0.94
	3	4.7	0.16	0.94
	4	4.9	0.13	0.98
	5	4.7	0.31	0.94
	6	4.8	0.16	0.96
Relevance	1	4.8	0.4	0.96
	2	4.8	0.4	0.96
	3	5	0	1
	4	5	0	1
	5	5	0	1
	6	5	0	1

Finally, the CVC validation was as follows:

- Appropriateness, defined as “The questions are easy to understand (clear, comprehensible for the level of information for a non-professional)” and “In the list, the questions are presented in a logical order”: CVC score 0.94.

- Relevance, defined as “The questions are relevant to achieving the instrument’s objective”: CVC score 0.99.

Based on the experts’ quantitative ratings and qualitative comments, the research team revised the wording and ordering of selected items while preserving the core domains identified during the conceptual development phase. The CVC stage was considered complete once all evaluated dimensions reached high CVC values, with appropriateness and relevance obtaining overall scores of 0.94 and 0.99, respectively. In addition, the qualitative comments provided by the experts did not indicate the need to remove any of the core domains or substantially modify the conceptual structure of the instrument. Therefore, after minor wording and ordering adjustments, the item pool was considered sufficiently clear, relevant, and conceptually stable to proceed to the formal CVR/CVI content validation stage.

#### 2.3.2 Formal content validation (CVR/CVI)

In this second stage, the aim was not to reassess wording or general comprehensibility, which had already been addressed during the CVC stage, but to determine whether each item was essential for representing the ecobiopsychosocial construct measured by AUTIVA. Thus, CVR was used to quantify expert agreement regarding item essentiality, while the CVI summarized the overall content validity of the complete instrument. After approval by the Ethics Committee and prior to implementation, AUTIVA underwent a formal content validation process using Lawshe’s Content Validity Ratio (CVR) (15) and the Content Validity Index (CVI). The CVR was calculated according to Lawshe’s original model, which is applicable to panels ranging from 9 to 40 experts. The formula used was: CVR = [ne − (N/2)] / (N/2).

where ne is the number of experts rating an item as essential and N is the total number of experts.

For this stage, 18 experts were consulted. The expert panel was intentionally multidisciplinary and included professionals from health, education, and social sciences, all with direct experience in autism assessment, intervention, education, or family support. Experts were selected according to their professional background, postgraduate training or specialized certification in autism or related fields, and practical experience working with autistic children and adolescents. To preserve confidentiality, individual names were not reported; however, the panel profile is summarized in Table 2. The group included professionals from pediatrics, psychology, occupational therapy, nutrition, special education, social work, and related areas. Their experience covered clinical assessment, therapeutic intervention, school inclusion, nutritional support, caregiver support, and public or community-based services for autistic children (See Table 2). Each expert rated every item using three response categories: “essential” for evaluating the construct, “useful but dispensable,” or “unnecessary.” For the CVR calculation, “essential” was coded as 1, while “useful but dispensable” and “unnecessary” were coded as 0.

**Table 2:** Profile of experts participating in the formal content validation process.

Expert area	Number of experts	Professional background	Academic/specialized training	Autism-related expertise
Health sciences	8	Pediatrics, psychology, occupational therapy, nutrition, and related clinical areas.	Postgraduate diploma, master’s degree, or specialized training in autism, child development, neurodevelopment, or rehabilitation.	Clinical assessment, therapeutic intervention, sensory integration, nutritional assessment, food selectivity, and family support.
Education	7	Special education, differential education, psychopedagogy, or school inclusion.	Postgraduate diploma, master’s degree, or specialized training in autism, inclusive education, or special educational needs.	School inclusion, educational support, curricular adaptations, classroom participation, and family-school coordination.
Social/community sciences	3	Social work, public health, community intervention, or related areas.	Specialized training in disability, autism, social protection, or community-based support.	Caregiver support, disability certification, socioeconomic barriers, service access, and public/community programs.
Total	18	Multidisciplinary panel	Autism-specific or related postgraduate/specialized training	Direct experience with autistic children, adolescents, families, or support systems

Following Lawshe’s original acceptance criterion, the minimum acceptable CVR value for a panel of 18 experts was set at 0.42. Items meeting or exceeding this threshold were considered to have sufficient expert agreement regarding their essentiality. Then, the CVI was calculated for the full instrument to estimate the overall degree of content validity across AUTIVA.

To provide a more cautious estimate of item-level content validity, 95% confidence intervals were calculated for each CVR. Since the CVR is a linear transformation of the proportion of experts rating an item as essential, where CVR = 2p − 1 and p = ne/N, confidence intervals were first estimated for the proportion of “essential” ratings using the Wilson score method and then transformed to the CVR scale. In addition, Wilson-adjusted CVR estimates were calculated by transforming the Wilson-adjusted proportion to the CVR scale. Lawshe’s original criterion for 18 experts, CVR ≥ 0.42, was retained as the primary decision rule for item retention (See Table 3).

**Table 3:** Raw and Wilson-adjusted CVR estimates according to the number of experts rating items as essential.

Experts rating the item as essential	Proportion rated as essential	Raw CVR	95% CI for CVR	Wilson-adjusted CVR	Number of items
18/18	1.00	1.00	0.65 to 1.00	0.82	46
17/18	0.94	0.89	0.48 to 0.98	0.73	9
16/18	0.89	0.78	0.34 to 0.94	0.64	4
15/18	0.83	0.67	0.22 to 0.88	0.55	1

CVR, Content Validity Ratio; CI, confidence interval; ne, number of experts rating the item as essential; N, total number of experts. Confidence intervals were calculated using the Wilson score method for proportions and transformed to the CVR scale. Lawshe’s original criterion for 18 experts, CVR ≥ 0.42, was used as the primary decision rule.

After completing the validation process, the survey was administered to primary caregivers of autistic children in the Los Ríos region. Statistical analyses were performed using SPSS version 22.0.

#### 2.3.3 Final AUTIVA instrument

The final AUTIVA instrument consists of 60 open-ended or multiple-choice questions and is composed of 6 subscales: General Pediatric Profile, Sex and Gender, Autism Diagnosis and Concomitant Pathologies, Family Income and the Impact of Autism on Socioeconomic Status, Diet of the Autistic Person, and Therapies and Schooling.

The first subscale, “General Pediatric Profile,” is composed of 13 questions, of which 3 are open-ended, 8 are dichotomous, one corresponds to the Bristol Scale (with pictures), and one requires anthropometry (weight, height, waist circumference and head circumference). This subscale seeks to assess sociodemographic data, biological, genetic and perinatal factors, family environment, lifestyles, primary care, and anthropometry. The latter was assessed by nutritionists trained in Autism.

The second subscale, “Sex and Gender,” consists of two questions, one of which is open-ended and the other is a nominal scale. This scale seeks to assess gender identity and expression, and identity trajectory.

The third subscale, “Autism Diagnosis and Concomitant Pathologies,” consists of seven questions, four of which are open-ended, one with nominal responses, and two with dichotomous responses. This scale seeks to assess clinical history and comorbidity, access to the health and social protection system, and level of functional support.

The fourth subscale, “Family Income and the Impact of Autism on Socioeconomic Status” consists of seven questions, two of which are open-ended, four with nominal responses, and one with dichotomous responses. This scale seeks to assess family structure, educational capital, family socioeconomic status, employment conditions, and the financial burden of the diagnosis.

The fifth scale, “Diet of the Autistic Person,” consists of 15 questions, 3 of which are open-ended and 12 with dichotomous responses. This scale seeks to identify eating patterns, autonomy, and sensory-nutritional profile.

The sixth scale, “Therapies and Schooling,” consists of 16 questions, 3 of which are open-ended, 3 with nominal scale responses, 6 with dichotomous responses, and 4 with Likert scale responses. This scale aims to gather comprehensive information on the therapeutic and educational trajectory of autistic children and adolescents.

## 3 Results

A total of 18 experts completed the content validation survey. The panel included professionals from pediatrics, psychology, education, occupational therapy, nutrition, and social sciences, all with extensive experience working with autistic children.

All 60 items exceeded Lawshe’s original minimum CVR criterion for a panel of 18 experts (CVR ≥ 0.42). Raw item-level CVR values ranged from 0.67 to 1.00. Most items showed very high agreement: 46 items obtained a CVR of 1.00, nine obtained a CVR of 0.89, four obtained a CVR of 0.78, and one obtained a CVR of 0.67. Wilson-adjusted CVR estimates ranged from 0.55 to 0.82. The lowest observed CVR value, corresponding to 15 of 18 experts rating the item as essential, was 0.67, with a Wilson-based 95% confidence interval of 0.22 to 0.88 and a Wilson-adjusted CVR of 0.55. These results support the retention of all items, while also indicating that the findings should be interpreted as initial expert-based evidence of content validity. (See Table 4).

**Table 4:** AUTIVA content validity ratio (CVR) for each subscale.

Subscale	Total questions per subscale	Response format	Content validity Ratio
General pediatric profile	13	3 open-ended 8 dichotomous responses 1 item for Bristol Scale (with pictures) 1 item concerning current anthropometry	0.94
Sex and gender	2	1 open-ended 1 nominal scale	0.89
Autism diagnosis and concomitant pathologies	7	4 open-ended 1 nominal response 2 dichotomous responses	0.98
Family income and the impact of autism on socioeconomic status	7	2 open-ended 4 nominal responses 1 dichotomous response	1.0
Diet of the autistic person	15	3 open-ended 12 dichotomous responses	0.96
Therapies and schooling	16	3 open-ended 3 nominal scale responses 6 dichotomous responses 4 Likert scale responses	1.0

The final content-validated version of AUTIVA includes six subscales: (1) General Pediatric Profile, (2) Sex and Gender, (3) Autism Diagnosis and Concomitant Pathologies, (4) Family Income and the Impact of Autism on Socioeconomic Status, (5) Diet of the Autistic Person, and (6) Therapies and Schooling.

## 4 Discussion

This study represents Phase I of a broader psychometric validation process. The aim of this phase was to develop AUTIVA and obtain initial expert-based evidence of content validity, rather than to establish the full psychometric validity of the instrument. First, AUTIVA underwent preliminary refinement using Hernández-Nieto’s CVC, with coefficients of 0.94 and 0.99 for appropriateness and relevance, respectively. Second, the instrument underwent formal content validation using Lawshe’s CVR and the CVI, obtaining a full-instrument CVI of 0.96. These findings indicate that experts considered the items relevant, understandable, and essential for representing the ecobiopsychosocial domains included in AUTIVA. However, they should not be interpreted as evidence of construct validity, criterion-related validity, internal consistency, or temporal stability. Rather, they establish a methodological foundation for the next phase of psychometric testing.

As previously stated, in Chile there are validated and standardized instruments for the autistic pediatric population that prioritize the detection of symptoms and description of behaviors, such as M-CHAT-R/F, AMSE and ADOS-2 (16–19). However, all of these instruments focus on early detection, screening, and supporting diagnostic confirmation through clinical observation and exclusively under a biomedical model.

Various studies have shown that many of the instruments used to assess people within the autism spectrum have significant limitations, as they tend to leave out key dimensions such as the social environment, environmental conditions and subjective experiences that directly affect their quality of life (20). Thus, a holistic and ecological perspective is currently required to complement existing instruments in order to expand the understanding of the multidimensionality of the autistic person (21, 22).

One of the central contributions of this instrument lies in its conception from the ecological perspective of human development (27 – 32). Moreover, this study also emphasizes the importance of interdisciplinarity (33 – 37), bringing together balanced perspectives that, based on disciplinary differences and experiences, allows for a deeper understanding of the autistic person (38 – 41), which represents an innovation from the assessment perspective. Furthermore, AUTIVA may provide policy makers with context-specific information on the needs of autistic children and their families. On this regard, current literature emphasizes the importance of having questionnaires or assessments specifically tailored to a particular population with a specific health situation (23, 24).

The AUTIVA instrument also had the active participation of autistic people and their families during its construction and implementation, which strengthens its sensitivity to capture subjective dimensions of the experience of this specific group of people (25), which strengthens its ecological, ethical and cultural validity (26). This bidirectional collaboration responds to the recommendations of participatory approaches in autism research, which have been shown to improve the relevance, applicability and acceptability of the tools developed (21).

The development of AUTIVA is also consistent with previous ecological and family-centered assessment frameworks used in child health, education and social care, such as the Common Assessment Framework and Early Help Assessment (27, 28). These approaches emphasize that children’s needs should be understood through the interaction between developmental characteristics, family functioning, environmental conditions and service responses. However, AUTIVA differs from these general child assessment frameworks by focusing specifically on autistic children and by incorporating domains that are particularly relevant to autism research and public health planning, including sensory-nutritional patterns, therapy trajectories, school inclusion, concomitant conditions, gender identity and the socioeconomic burden of care.

Furthermore, its in-person application is noteworthy, allowing for direct contact with the person being assessed, incorporating contextual observations, such as nutritional assessment, which is rarely considered by current instruments, and often relies on self-reporting (29).

Also, this instrument is of global significance because it provides a comprehensive assessment framework that can be adapted to diverse contexts, especially as autism prevalence continues to rise worldwide.

Construct validity, criterion-related validity, and reliability were not evaluated in the present study, as these analyses were beyond the scope of this initial content validation phase. Although the six subscales of AUTIVA were theoretically derived from the ecobiopsychosocial framework and refined through participatory and expert-based procedures, future studies must examine whether this proposed structure is empirically supported. This should include exploratory and/or confirmatory factor analyses, depending on sample size, item distribution, and the measurement level of each item. However, given that AUTIVA includes open-ended, dichotomous, nominal, ordinal, Likert-type, and anthropometric items, future construct validity analyses may require mixed psychometric strategies rather than relying exclusively on traditional factor analytic approaches. Reliability should also be assessed according to the nature of each domain and item format. Internal consistency indices, such as Cronbach’s alpha or McDonald’s omega, may be appropriate only for groups of items intended to measure a common latent construct, whereas dichotomous or categorical items may require alternative approaches, such as Kuder–Richardson coefficients, inter-rater agreement, or stability estimates. Test–retest reliability should also be examined to assess the temporal stability of caregiver responses, particularly for domains related to service access, therapy participation, school inclusion, and family socioeconomic burden. In parallel, future research should examine criterion-related validity by assessing the association between AUTIVA domains and external criteria relevant to autism-related public health assessment, including adaptive functioning, quality of life, caregiver burden, nutritional status, therapy access, school participation, educational support needs, disability certification, and service-use indicators. These analyses will help determine whether AUTIVA domains are empirically coherent, reliable over time, and meaningfully associated with established measures or relevant public health outcomes.

Finally, this instrument does not seek to identify deficits, but rather to comprehensively map both the barriers and strengths of each autistic person within their ecosystem, which aligns with recent proposals for assessment models focused on well-being and self-determination (30, 31). Furthermore, it advocates a rights-based approach that recognizes gender diversity in autistic individuals, opening up a new and previously unaccounted-for perspective within this population in Chile. It notes that the expression of autism can be influenced by factors such as gender and culture, as well as physical and behavioral characteristics (32), which are not always considered in screening and diagnostic instruments. This limitation could affect the accuracy of current estimates of autism prevalence and the targeting of specific interventions based on the profile in different participation contexts.

## 5 Conclusion

This study presents the development and content validation of AUTIVA, a multidimensional ecobiopsychosocial instrument designed to assess autistic children within the Chilean context. Expert evaluation demonstrated strong agreement across all items and subscales, providing initial expert-based evidence of the instrument’s content relevance, representativeness, and conceptual coherence. AUTIVA addresses a critical gap in existing assessment tools by integrating clinical, nutritional, educational, therapeutic, socioeconomic, and environmental factors that shape the experiences of autistic children and their families, in order to create or assess public policies.

While additional psychometric analyses are required to evaluate reliability, construct validity, criterion-related validity, and applicability in diverse populations, the present findings establish a solid foundation for future testing and implementation. AUTIVA has the potential to support interdisciplinary assessment, inform research and public health planning, and contribute to more contextually grounded and equitable approaches to autism evaluation in Latin America.

### 5.1 Future research

Future research should conduct the next phase of AUTIVA’s psychometric evaluation in a larger and more diverse sample of primary caregivers of autistic children and adolescents. This phase should examine construct validity by testing whether the proposed subscales are empirically supported, using exploratory and/or confirmatory factor analyses when appropriate. Because AUTIVA includes multiple response formats—including open-ended, dichotomous, nominal, ordinal, Likert-type, and anthropometric items—future validation should consider mixed psychometric strategies rather than relying exclusively on internal consistency estimates. Reliability should be assessed through test–retest procedures and, where applicable, internal consistency indices such as Cronbach’s alpha, McDonald’s omega, or coefficients suitable for dichotomous items. Criterion-related validity should also be examined by comparing AUTIVA domains with external indicators such as adaptive functioning, quality of life, caregiver burden, nutritional status, educational support needs, therapy access, disability certification, and service-use measures. Cross-cultural validation in other Latin American countries would further strengthen its applicability.

### 5.2 Strengths and limitations

A major strength of this study is the inclusion of a diverse panel of experts with direct experience in autism assessment and care, which enhances the ecological validity of the content evaluation. The participatory approach during item development also ensured that widely reported but undermeasured contextual factors—such as therapy affordability, nutritional challenges, and caregiving strain—were incorporated into the instrument. However, this study has several limitations. First, the validation process focused exclusively on content validity; therefore, AUTIVA should not yet be considered a fully validated psychometric instrument. Evidence of construct validity, criterion-related validity, internal consistency, test–retest reliability, and measurement invariance remains necessary. Second, although the six subscales were theoretically derived from an ecobiopsychosocial framework and refined through participatory and expert-based procedures, their empirical structure has not yet been tested. Future studies should examine the dimensionality of AUTIVA using exploratory and/or confirmatory approaches, when appropriate, while considering the heterogeneous format of its items. Third, criterion-related validity was not assessed in this phase; future research should compare AUTIVA domains with external indicators such as adaptive functioning, quality of life, caregiver burden, nutritional status, educational support needs, disability certification, and service-use measures. Fourth, reliability analyses were not conducted. Future work should assess internal consistency only for item groups intended to measure common latent constructs, while using alternative reliability approaches for dichotomous, categorical, open-ended, and anthropometric items. Finally, the instrument was developed within the Chilean context, which may limit generalizability and requires cross-cultural adaptation before use in other Latin American countries.

## Data Availability

The original contributions presented in the study are included in the article/Supplementary material, further inquiries can be directed to the corresponding author.

## References

[1] Alcala GC Ochoa MG. Trastorno del espectro autista (TEA). Rev Fac Med (Méx). (2022) 65:7–20. doi: 10.22201/fm.24484865e.2022.65.1.02

[2] World Health Organization. Autism. Geneva: World Health Organization (2025).

[3] Oyarzún Gómez D Casas Aznar F Alfaro Inzunza J. Family, school, and neighbourhood microsystems influence on children’s life satisfaction in Chile. Child Indic Res. (2019) 12:1915–33. doi: 10.1007/s12187-018-9617-5

[4] Carrillo S Ripoll-Núñez K. "Social development of children living in poverty in Colombia: an analysis from an integrative perspective". In: Rosabal-Coto M Tapia-Balladares J, editors. Family and Contexts of Development: Challenges in Latin America. New York: Oxford University Press (2025). p. 18–35.

[5] Barcelata Eguiarte BE Suárez BP. "An ecological-systemic framework: an overview of child and adolescent development in adverse contexts". In: Barcelata Eguiarte BE Suárez Brito P, editors. Child and Adolescent Development in Risky Adverse Contexts: a Latin American Perspective. Cham: Springer (2021). p. 3–22.

[6] Ministerio de Educación de Chile. Preguntas frecuentes Ley TEA 21.545. Santiago: Ministerio de Educación de Chile (2025).

[7] Yáñez C Maira P Elgueta C Brito M Crockett M Troncoso L . Prevalence estimation of autism spectrum disorders in Chilean urban population. Andes Pediatr. (2021) 92:519–25. doi: 10.32641/andespediatr.v92i4.2503 34652369

[8] Engel GL. The need for a new medical model: a challenge for biomedicine. Science. (1977) 196:129–36. 847460 10.1126/science.847460

[9] Ministerio de Salud de Chile. Manual de calificación y certificación de discapacidad. Santiago: Ministerio de Salud de Chile (2022).

[10] Earls F Carlson M. The social ecology of child health and well-being. Annu Rev Public Health. (2001) 22:143–66. doi: 10.1146/annurev.publhealth.22.1.143, 11274516

[11] Ministerio de Educación de Chile Ley de inclusión escolar Santiago Ministerio de Educación de Chile (2025) Available online at: https://leyinclusion.mineduc.cl/ley-de-inclusion/ (Accessed December 15, 2025).

[12] Biblioteca del Congreso Nacional de Chile. Ley 21545: establece la promoción de la inclusión, la atención integral, y la protección de los derechos de las personas con trastorno del espectro autista en el ámbito social, de salud y educación. Santiago: Biblioteca del Congreso Nacional de Chile (2023).

[13] Echeita-Sarrionandia G. Educación inclusiva: el sueño de una noche de verano. 2nd ed. Barcelona: Octaedro (2019). p. 50–9.

[14] Pedrosa I Suárez-Álvarez J García-Cueto E. Evidencias sobre la validez de contenido: avances teóricos y métodos para su estimación. Accion Psicol. (2013) 10:3–18. doi: 10.5944/ap.10.2.11820

[15] Gilbert GE Prion S. Making sense of methods and measurement: Lawshe’s content validity index. Clin Simul Nurs. (2016) 12:530–1. doi: 10.1016/j.ecns.2016.08.002

[16] Lord C Rutter M DiLavore PC Risi S Gotham K Bishop SL. ADOS-2. Escala de observación para el diagnóstico del autismo-2: manual, adaptación española. Madrid: TEA Ediciones (2015). p. 17–47.

[17] Irarrázaval M López I Figueroa C Cabezas M Yáñez C Rodillo E . Adaptación y validación del Examen de Estado Mental del Autismo (AMSE) en Chile: buscando reducir la brecha diagnóstica. Andes Pediatr. (2023) 94:475–84. doi: 10.32641/andespediatr.v94i4.4476, 39808557

[18] Gatica-Bahamonde G Méndez-Fadol A Sánchez-Sepúlveda F Peñailillo-Díaz C van Kessel R Czabanowska K . Testing an online screening for autism in the COVID-19 pandemic: a psychometric study of the Q-CHAT-24 in Chilean toddlers. Front Psychol. (2024) 15:1363976. doi: 10.3389/fpsyt.2024.1363976, 38952633 PMC11215167

[19] Okoye C Obialo-Ibeawuchi CM Obajeun OA Sarwar S Tawfik C Waleed MS . Early diagnosis of autism spectrum disorder: a review and analysis of the risks and benefits. Cureus. (2023) 15:e43226. doi: 10.7759/cureus.43226, 37692637 PMC10491411

[20] Crane L Adams F Harper G Welch J Pellicano E. “Something needs to change”: mental health experiences of young autistic adults in England. Autism. (2019) 23:477–93. doi: 10.1177/1362361318757048, 29415558

[21] Fletcher-Watson S Adams J Brook K Charman T Crane L Cusack J . Making the future together: shaping autism research through meaningful participation. Autism. (2019) 23:943–53. doi: 10.1177/1362361318786721, 30095277 PMC6512245

[22] Fletcher-Watson S Happé F. Autism: a new Introduction to Psychological Theory and current Debate. 2nd ed. London: Routledge (2020). p. 154–96.

[23] Mosquera-Nogueira J Rodríguez-Míguez E. Measurement of quality of life in primary care. Cad Aten Primaria. (2020) 26:23–8.

[24] Alarcón-Rodríguez AA Carreño-Moreno SP Arias-Rojas M. Validez y confiabilidad del instrumento Carga de la Enfermedad Crónica del Paciente GCPC-UN. Av Enferm. (2020) 38:296–306. doi: 10.15446/av.enferm.v38n3.84031

[25] den Houting J. Neurodiversity: an insider’s perspective. Autism. (2019) 23:271–3. doi: 10.1177/1362361318820762, 30556743

[26] Nicolaidis C Raymaker DM McDonald KE Lund EM Boisclair WC Beers LM . Creating accessible research training curricula for autistic people and people with intellectual disability. J Empir Res Hum Res Ethics. (2020) 15:172–83. doi: 10.1177/1556264620919115

[27] Moscon V. Enhancing public sector performance: a systematic review of the common assessment framework. Int J Bus Res Manag. (2025) 16:44–63.

[28] Lucas S Archard PJ. Early help and children’s services: exploring provision and practice across English local authorities. J Child Serv. (2021) 16:74–86. doi: 10.1108/JCS-03-2020-0009

[29] Ratto AB Kenworthy L Yerys BE Bascom J Wieckowski AT White SW . What about the girls? Sex-based differences in autistic traits and adaptive skills. J Autism Dev Disord. (2018) 48:1698–711. doi: 10.1007/s10803-017-3413-9, 29204929 PMC5925757

[30] Kapp SK. Autistic Community and the Neurodiversity Movement: Stories from the Frontline. Singapore: Palgrave Macmillan (2020). p. 141–3.

[31] Milton D. On the ontological status of autism: the “double empathy problem”. Disabil Soc. (2012) 27:883–7. doi: 10.1080/09687599.2012.710008

[32] McNealis M Kent J Paskov K Dunlap K Lane J Phillips B . Identifying understudied correlations between autism and phenotypic attributes in a large family dataset. BMC Psychol. (2025) 13:561. doi: 10.1186/s40359-025-02739-4, 40420291 PMC12105231

[33] Coelho-Medeiros ME Bronstein J Aedo K Pereira JA Arraño V Pérez CA . Validación del M-CHAT-R/F como instrumento de tamizaje para detección precoz en niños con trastorno del espectro autista. Rev Chil Pediatr. (2019) 90:492–9. doi: 10.32641/rchped.v90i5.703, 31859732

[34] Milliron BJ Zegans M Deutsch J. Creating a more powerful framework for research, teaching, and health promotion: an eco-biopsychosocial model. Acta Hortic. (2021) 1330:261–8. doi: 10.17660/ActaHortic.2021.1330.31

[35] Wilson DK Lawman HG. "Health promotion in children and adolescents: an integration of the biopsychosocial model and ecological approaches to behavior change". In: Roberts MC Steele RG, editors. Handbook of Pediatric Psychology, 4th Edn. New York: The Guilford Press (2009). p. 603–17.

[36] Milliron BJ Zegans M Deutsch JM. Toward an eco-biopsychosocial framework for understanding food, nutrition, and health: the crucial role of food studies and food systems. Nutr Health. (2025) 31:1779–85. doi: 10.1177/02601060251324241, 40094807

[37] Rosa EM Tudge J. Urie Bronfenbrenner’s theory of human development: its evolution from ecology to bioecology. J Fam Theory Rev. (2013) 5:243–58. doi: 10.1111/jftr.12022

[38] Schalock RL Keith KD Verdugo M Gómez LE. "Quality of life model development and use in the field of intellectual disability". In: Kober R, editor. Enhancing the Quality of life of People with Intellectual Disabilities. Dordrecht: Springer (2010). p. 17–32.

[39] Morán-Suárez ML Gómez-Sánchez LE Alcedo-Rodríguez MA. Social inclusion and self-determination: the challenges in the quality of life of youth with autism and intellectual disability. Siglo Cero. (2019) 50:29–46. doi: 10.1016/j.ridd.2025.105071

[40] Verdugo-Alonso MA Schalock RL. Changes in the understanding and approach to persons with intellectual disability. Siglo Cero. (2010) 41:7–21.

[41] Bronfenbrenner U. La ecología del desarrollo humano: experimentos en entornos naturales y diseñados. 1st ed. Barcelona: Paidós Ibérica (1987). p. 25–34.

[42] Andrés-Villas M Torrico-Linares ME Santín-Vilariño MC Menéndez Álvarez-Dardet S López-López MJ. El modelo ecológico de Bronfenbrenner como marco teórico de la psicooncología. An Psicol. (2002) 18:45–59.

[43] Fiallo J. La interdisciplinariedad en la escuela: un reto para la calidad de la educación. 1st ed. Ciudad de La Habana: Editorial Pueblo y Educación (2011). p. 74–7.

[44] Bell Rodríguez RF Orozco Fernández II Lema Cachinell BM. Interdisciplinariedad, aproximación conceptual y algunas implicaciones para la educación inclusiva. Episteme. (2022) 9:101–16.

[45] Kunze M Machalicek W. Interdisciplinary teams: a model to support students with autism. Psychol Sch. (2022) 59:1350–62. doi: 10.1002/pits.22618

[46] Rosenau KA Hotez E. Promoting interdisciplinary and participatory autism research. Pediatrics. (2022) 149:e2020049437P. doi: 10.1542/peds.2020-049437P, 35363283

[47] Bowman KS Suarez VD Weiss MJ. Standards for interprofessional collaboration in the treatment of individuals with autism. Behav Anal Pract. (2021) 14:1191–208. doi: 10.1007/s40617-021-00560-0, 34868822 PMC8586309

[48] Ministerio de Educación de Chile. Orientaciones técnicas para programas de integración escolar (PIE). Santiago: Ministerio de Educación de Chile (2013).

[49] Basurto-Álava PS Loor-Zambrano DL Bravo-Sánchez RE Cantos-Ventura XM Rodríguez-García MA. La interdisciplinariedad y la multidisciplinariedad en el contexto educativo postpandemia. Polo Conoc. (2023) 8:2487–504. doi: 10.23857/pc.v8i8.5982

[50] Henao-Villa CF García-Arango DA Aguirre-Mesa ED González-García A Bracho-Aconcha R . Multidisciplinariedad, interdisciplinariedad y transdisciplinariedad en la formación para la investigación en ingeniería. Rev Lasallista Investig. (2017) 14:179–97. doi: 10.22507/rli.v14n1a16

[51] Paoli-Bolio FJ. Multi, inter y transdisciplinariedad. Probl Anu Filos Teor Derecho. (2019) 13:347–57. doi: 10.22201/iij.24487937e.2019.13

