# Supplementary material for: Design and content validation of AUTIVA: an ecobiopsychosocial instrument for childhood autism
Source: Front Public Health. 2026 Jul 22;14:1768438. doi: 10.3389/fpubh.2026.1768438 (PMC13438446; doi:10.3389/fpubh.2026.1768438)
Supplement: Supplementary file 1 [file Data_sheet_1.docx]

**Supplementary Material S1. Description of the AUTIVA Instrument Subscales**

**S1.1. General Pediatric Profile**

The first 13 questions of the “General Pediatric Profile” subscale refer to: date of birth, gestational age at birth, identification of the primary caregiver, consanguinity between parents, first-degree relative diagnosed with autism, vaccination status, physical activity, prescribed medications, updated anthropometry (weight, height, waist circumference and head circumference), sphincter control and description of bowel movements (with an image of the Bristol scale).

**S1.2. Sex and Gender**

The next two questions in the "Sex and Gender" subscale address: gender of identification and if there is disagreement with the sex assigned at birth at what did it occur.

**S1.3. Autism Diagnosis and Concomitant Pathologies**

The next seven questions in the "Autism Diagnosis and Concomitant Pathologies" subscale address: age of first signs of autism and which signs were present, age at which autism was diagnosed, level of support required by the autistic child (Level 1: Requiring Support, Level 2: Requiring Substantial Support, and Level 3: Requiring Very Substantial Support), whether the child is listed in the National Disability Registry, and the presence of the most common concomitant pathologies (intellectual disability, epilepsy, sensory integration disorder, sleep disorder, motor dyspraxia, hearing impairment, behavioral disorders, and others).

**S1.4. Family Income and Socioeconomic Impact**

The following 7 questions in the subscale “Family Income and the Impact of Autism on Socioeconomic Status” refer to: number of people in the family unit, education level of the primary caregiver, total monthly family income, money allocated to ASD treatment (medications, therapies, others), type of work and remuneration of the primary caregiver, and self-care activities of the primary caregiver.

**S1.5. Diet Subscale**

The following 15 questions in the “Diet of the autistic person” subscale refer to: person that prepares the food for the autistic child, person that feeds the autistic person, presence of food allergies, consumption of fruits, vegetables, dairy products, meat products (beef, fish), legumes, ultra-processed foods (fried foods, sweet or salty snacks, sugary drinks), water, and food color preference.

**S1.6. Therapies and Schooling**

The last 16 questions of the "Therapies and Schooling" subscale refer to: time dedicated to and type of therapy received by the autistic child or adolescent, age at which first therapy associated with autism was received, professionals involved in the diagnosis (occupational therapist, speech therapist, kinesiologist, neurologist, psychiatrist, nutritionist, educational psychologist, special educator, psychologist), family integration and participation in therapeutic treatment, progress of the autistic person through therapy. Schooling of the child or adolescent (type of establishment, type of financing and current school year), relationship with classmates, participation in activities inside and outside the classroom (free-choice curricular activities, extracurricular activities, School Integration Program), use of sensory adaptation in classes, request for neurological/psychiatric evaluation by the educator, perception of support received by the classroom team, progress of the child or adolescent within the educational system, bullying and suspension/expulsion measures for the autistic child or adolescent.

This document accompanies the article “Design and Content Validation of AUTIVA” submitted to Applied Sciences and provides additional detail on the content of each instrument subscale.
